# Chromatin accessibility analysis identifies *GSTM1* as a prognostic marker in human glioblastoma patients

**DOI:** 10.1186/s13148-021-01181-8

**Published:** 2021-11-03

**Authors:** Yin-Cheng Huang, Joseph Chieh-Yu Lai, Pei-Hua Peng, Kuo-Chen Wei, Kou-Juey Wu

**Affiliations:** 1 Department of Neurosurgery, Chang Gung Memorial Hospital at Linkou, Taoyuan, 333 Taiwan; 2 Institute of Biomedical Science, China Medical University, Taichung, 404 Taiwan; 3 Cancer Genome Research Center, Chang Gung Memorial Hospital at Linkou, Gueishan District, Taoyuan, 333 Taiwan; 4 Institute of Cellular and Organismic Biology, Academia Sinica, Taipei, 115 Taiwan; 5 Graduate Institute of Clinical Medical Sciences, Chang Gung University, Taoyuan, 333 Taiwan; 6 Department of Medicine, Chang Gung University, Taoyuan, 333 Taiwan

**Keywords:** Glioblastomas, Next generation sequencing, ATAC-seq, Chromatin accessibility, RNA-seq, GSTM1, STAT3

## Abstract

**Background:**

Glioblastoma (GBM) is a malignant human brain tumor that has an extremely poor prognosis. Classic mutations such as *IDH* (isocitrate dehydrogenase) mutations, *EGFR* (epidermal growth factor receptor) alternations, and *MGMT* (O6-methylguanine-methyltransferase) promoter hypermethylation have been used to stratify patients and provide prognostic significance. Epigenetic perturbations have been demonstrated in glioblastoma tumorigenesis. Despite the genetic markers used in the management of glioblastoma patients, new biomarkers that could predict patient survival independent of known biomarkers remain to be identified.

**Methods:**

ATAC-seq (assay for transposase accessible chromatin followed by sequencing) and RNA-seq have been used to profile chromatin accessible regions using glioblastoma patient samples with short-survival versus long-survival. Cell viability, cell cycle, and Western blot analysis were used to characterize the cellular phenotypes and identify signaling pathways.

**Results:**

Analysis of chromatin accessibility by ATAC-seq coupled with RNA-seq methods identified the *GSTM1* (glutathione S-transferase mu-1) gene, which featured higher chromatin accessibility in GBM tumors with short survival. GSTM1 was confirmed to be a significant prognostic marker to predict survival using a different GBM patient cohort. Knockdown of *GSTM1* decreased cell viability, caused cell cycle arrest, and decreased the phosphorylation levels of the NF-kB (nuclear factor kappa B) p65 subunit and STAT3 (signal transducer and activator of transcription 3) (pSer727).

**Conclusions:**

This report demonstrates the use of ATAC-seq coupled with RNA-seq to identify *GSTM1* as a prognostic marker of GBM patient survival. Activation of phosphorylation levels of NF-kB p65 and STAT3 (pSer727) by GSTM1 is shown. Analysis of chromatin accessibility in patient samples could generate an independent biomarker that can be used to predict patient survival.

**Supplementary Information:**

The online version contains supplementary material available at 10.1186/s13148-021-01181-8.

## Introduction

Glioblastoma (GBM) remains a devastating disease with a median survival of 13–16 months despite advances in surgery, radiation therapy, chemotherapy and targeted therapy [1–3]. The classifications of primary glioblastomas are based on classic mutations, including *IDH* mutations, 1p/19q codeletion, *MGMT* promoter methylation, *G-CIMP* (glioma-CpG island methylator phenotype) methylation, *TERT* (telomerase reverse transcriptase) promoter mutations, *EGFR* genomic alterations/mutations, LOH (loss of heterozygosity) at 10q, *p16/INK4A* deletion, and *PTEN* (phosphatase and tensin homolog) mutations [4–8]. These classic mutations can be used to provide prognostic implications. For example, *EGFR* alterations and *TERT* promoter mutations are associated with poor prognosis, whereas *IDH* mutations and *MGMT* promoter methylation are associated with favorable prognosis in glioblastoma patients [6–9].

Epigenetic alterations or dysfunctions in glioblastoma involves three levels, including DNA methylation, histone modifications, and chromatin remodeling [10, 11]. Whole genome sequencing showed the frequent incidence of histone H3 mutations in pediatric glioma patient samples and aberrant expression of *EZH2* (enhancer of zeste homolog 2) and *KDMs* (lysine demethylases) in GBM patient samples [7, 10, 11]. *HDACs* (histone deacetylases) overexpression have also been shown to confer therapeutic resistance and tumor growth in glioblastoma [11, 12]. Therefore, EZH2 inhibitors, KDM inhibitors, and HDAC inhibitors may serve as therapeutic agents for GBM patients, although some of the inhibitors did not have satisfying results [10–12].

ATAC-seq (assay for transposase-accessible chromatin followed by sequencing) coupled with RNA-seq could be used in the profiling of chromatin accessibility regions in different types of human cancers to generate prognostic markers [13, 14]. For example, utilizing ATAC-seq to explore the differences in chromatin accessibility between neural stem cells and tumor-specific/migratory stem cells, TEAD1 (TEA domain family member 1) has been identified as a regulator of migration in glioblastoma [15].

Glutathione S-transferase mu-1 (GSTM1) is a member of the glutathione-S transferase (GST) family, which detoxifies by conjugating reduced glutathione (GSH) to target proteins in different organs [16]. GSTs could play a role in drug resistance through direct detoxification or involvement in the MAP (mitogen-activated protein) kinase pathway [17]. GSTM1 is one of the most abundant proteins in astrocytes [18]. In contrast to the association between *GSTM1* polymorphism and tumorigenesis in other tumor types, there is no association between *GSTM1* polymorphism and adult glioma [19]. Deletion of *GSTM1* has been shown to be associated with pediatric glioma [20]. In mechanistic studies, GSTM1 has been shown to promote inflammatory signaling through activation of NF-kB and production of GM-CSF (granulocyte–macrophage colony stimulating factor) and CCL2 (chemokine [C–C motif] ligands 2) in astrocytes during brain inflammation [21]. GSTM1 also modulates allergen-induced NF-kB activation in asthmatic airway epithelium [22]. Although NF-kB signaling has been shown to promote gliomagenesis [23], the role of GSTM1 in enhancing NF-kB activity in glioblastoma remains to be determined. Regarding other signaling pathways involved in GBM, STAT3 has been shown to be a molecular hub and its role in GBM tumor progression has been demonstrated [24, 25]. However, the role of GSTM1 in activating STAT3 signaling also remains to be determined.

To compare the chromatin accessibility landscape between short-term (< 0.5 year) and long-term (> 2 year) surviving GBM sample types for the discovery of novel biomarkers, ATAC-seq coupled with RNA-seq from these two different GBM sample types was performed. Among the genes upregulated in GBM samples with short survival, *GSTM1* was selected for further analysis due to its high hazard ratio and possible role in regulating signaling pathways (NF-kB, STAT3) involved in gliomagenesis [23–25]. Our results confirmed *GSTM1* as a biomarker that could predict the survival of GBM patients. The STAT3 signaling pathway was also shown to be regulated by GSTM1. This report demonstrates that analysis of the chromatin accessibility landscape can lead to the discovery of a useful biomarker that predicts the survival of GBM patients.

## Material and methods

### Clinical samples collection

The patient samples were collected from tissues cryo-preserved by liquid nitrogen from the tissue bank of Chang Gung Memorial Hospital at Linkou and they were confirmed WHO grade IV glioblastoma samples (Table 1). The first set of 10 glioblastoma tissue (5 short-term survival and 5 long-term survival patients) were obtained for gene analysis (IRB consent, CGMH-201900138B0). These samples were selected based on the survival duration. From our archives, the short-term survival was defined as less than 0.5 years and long-term survival was defined as longer than 2 years. Patients with short-term or long-term survival reflected the chemoresistant (CR) or chemosensitive (CS) status, respectively. For abbreviation, the terminology of “short-survival” and “long-survival” will be used. The median age was 58 (ranged from 41 to 72, mean = 58.3). Seven patients were male and three were female. The tissue was stored in liquid nitrogen immediately after surgical resection. The DNA/RNA extraction and library construction were performed by standard protocols. For sequencing, we selected 2 normal tissues and 4 tumor tissues of patients with long survival and 4 tumor tissues of patients with short survival for RNA-seq; 4 normal tissues, 4 tumor tissues of patients with long survival and 4 tumor tissues of patients with long survival for ATAC-seq (the detailed sequencing information was shown in Table 1). The second cohort was 125 paraffin-embedded grade IV glioblastoma samples which were also obtained from the tissue bank of Chang Gung Memorial Hospital under IRB approval (CGMH-201900138B0). It was an independent cohort and the tumor tissues were collected consecutively from the neurosurgical department archives without any selection. These samples were used for immunohistochemistry (IHC) staining.

**Table 1:** RNA-seq and ATAC-seq patient profile

Patient profile	Tissue ID	Overall survival (days)	Onset age	Sex	ATAC-seq	RNA-seq
Short-survival (chemoresistant; CR) (< 0.5 years)	Short-Survival-1	145	58	M	v	v
	Short-Survival-2	147	72	F	v	v
	Short-Survival-3	173	58	M	-	v
	Short-Survival-4	183	68	F	v	v
	Short-Survival-5	112	41	M	v	–
Long-survival (chemosensitive; CS) (> 2 years)	Long-survival-1	1500	58	M	v	v
	Long-survival-2	2633	57	M	-	v
	Long-survival-3	1037	61	M	v	v
	Long-survival-4	1461	54	M	v	v
	Long-survival-5	1597	54	F	v	–
Normal	Normal-1		63	F	v	v
	Normal-2		54	M	v	v
	Normal-3		53	M	v	–
	Normal-4		65	M	v	–

### ATAC-seq on frozen tissue

Omni-ATAC was performed for gathering tissue ATAC-seq data by following the previous study [14]. Frozen tissue was thawed into 1 ml iced cold HB buffer (320 mM sucrose, 0.1 mM EDTA, 0.1% NP40, 5 mM CaCl2, 3 mM Mg(Ac)2, 10 mM Tris pH 7.8, 1 × protease inhibitors (Roche, cOmplete), and 167 μM β-mercaptoethanol in ultrapure water) inside 2 ml Dounce homogenizer. The thawed frozen tissue was homogenized with pestle A (loose) for 10 times stroke, followed by filtering homogenized sample with 100 μm nylon mesh. The filtered sample was homogenized again with pestle B (tight) for 20 times stroke. After releasing the nuclei by douncing, 400 ul of homogenized tissue was mixed with 400 ul of 50% iodixanol (inside 1 × homogenization buffer) followed by pipetting, making the final 25% gradient solution with a total volume of 800 ul of homogenized tissue. 29% iodixanol and 35% iodixanol solution was prepared and layered underneath the 25% gradient solution that contained homogenized tissue. The sample was centrifuged in swing-bucket 3000 g for 30 min at 4 °C to form a nuclei band that stayed at the interface between 29 and 35% iodixanol solutions. 300 μl of solution containing nuclei was collapsed followed by gathering 5 × 10^4^ counted nuclei into 1 ml ATAC-RSB buffer (10 mM Tris–HCl pH 7.4, 10 mM NaCl, and 3 mM MgCl_2_ in ultrapure water) with 0.1% Tween-20. The nuclei were pelleted by centrifugation at 500 g for 5 min at 4 °C. After removing the supernatant, transposase mixture (25 μl 2 × TD buffer, 2.5 μl transposase (100 nM final), 16.5 μl PBS, 0.5 μl 1% digitonin, 0.5 μl 10% Tween-20, 5 μl ultrapure water) was directly added into nuclear pellet followed by pipetting 6 times. Transposase reaction was performed at ThermoMixer 1000 rpm for 30 min at 37 °C. Transposed DNA was collapsed followed by Zymo DNA Clean and Concentrator. Library was constructed by PCR amplification with NEB Next High-Fidelity PCR mix according to previous method [26]. The amplified library was purified by 1.8X Ampure XP beads clean-up through following the manufacture’s protocol. Constructed library was quality-controlled by Tapstation to avoid primer dimer, then sequenced by NextSeq 550 with 75 bp paired-end sequence.

### RNA extraction from frozen tissue and RNA-seq

Frozen tissue was homogenized by MagNA Lyser Green Beads (03358941001, Roche). After homogenization, RNA was extracted by RNeasy according to the manufacturer’s protocol. RNA RIN score was checked by RNA screen tape with Tapstation (Agilent). RNA-seq library was constructed using the KAPA RiboMinus kit under stranded library construction. Library was sequenced by Illumina Nextseq 550 150 bp paired-end sequence.

### Sequence data analysis

ATAC-seq data were aligned to hg38 by bowtie2 [27]. After the alignment, files were filtered according to the mapping quality and the PCR duplicates were removed. Chromatin accessible regions were called by MACS [28]. Differential chromatin accessibility was performed by diffbind, R package [29]. RNA-seq data were aligned to hg38 by hisat2 [30]. After the alignment, files were filtered according to the mapping quality, and the abundance of gene expression by ht-seq were counted. Differential gene analysis was performed by edgeR [31]. For further comprehensive analysis, peak sets from diffbind results were annotated by ChIPSeeker, a R package with Gencode v31 [32]. Promoter was defined to be -3000 to 500 bp surrounding the transcription start sites of each transcript. Under the consideration of accessible region located in the promoter that correlated with gene activation, we selected promoter peak sets that correlated with the differential results and linked the accessible region to gene expression by the coordinate of each gene.

### Quality control of clinical sample

ATAC-seq library were quality-controlled by tapstation before sequencing. TSS enrichment score were calculated for data filtering (TSS enrichment score > 1) and principal component analysis (PCA) was performed during the bioinformatics processing. Before RNA-seq library construction, extracted RNAs were quality-controlled by RNA RIN value with tapstation. Samples with RIN value above 4 were selected for library construction followed by sequencing.

### Immunohistochemistry (IHC) staining

The IHC staining of paraffin-embedded tissues of the independent cohort that contained 125 paraffin-embedded GBM samples was performed as previously described [33]. The tissue (4–5 μM in thickness) was mounted onto slides. The tissue was deparaffinized and rehydrated. Antigen retrieval was performed with citrate buffer and boiled at 100 °C for 10 min. The primary antibody was diluted as suggested (GSTM1, 1H4F2, Novus Biologicals, NBP2-22186) at 4 °C overnight and washed for 3 times. Then secondary antibody was incubated for 1 h at room temperature. After another 3 washes, DAB (3,3’-diaminodbenzidine) chromogenic was applied, followed by hematoxylin counterstain (Thermo-Fisher Scientific Inc. UltraVision Quanto Detection System, TL-125-QHD).

### IHC scoring

A scoring system for IHC was performed as described, from 0 to 3+ [34]. To further categorize the heterogeneous expression of DAB chromogen in the tissue, the scoring system of 0–1+ was defined as low-expression and 2+ to 3+ as high-expression. The personnel who scored the level of expression was an experienced neurosurgeon with extensive laboratory experience.

### Survival analysis

A Kaplan–Meier survival analysis, non-parametric statistics, was used to estimate the survival functions of high and low expression of target genes. Other factors, such as sex, age, *MGMT* (O6-methylguanine-methyltransferase) status and *IDH1* (isocitrate dehydrogenase 1) mutation was analyzed with log-rank and Wilcoxon test. The person who scored the expression of IHC staining was blind to the survival data of the patients.

### Expression of *GSTM1* by quantitative RT-PCR (qRT-PCR)

The quantitative real-time PCR (qRT-PCR) was used to quantify the expression of *GSTM1* with SYBR-Green system. Primers for *GSTM1* and *β-actin* was designed by Primer 3 (*GSTM1*; forward: AGCGGCCATGGTTTGCAGGAA, reverse: TTCTCCAAGCCCTCAAAGCGG). Briefly, the cDNA triplicate experiments were amplified at 95 °C for 1 min, followed by denaturing, annealing and extension cycles in SYBR Green master mix (Invitrogen) with LightCycler 480 (Roche Molecular Systems, Inc). The expression level is calculated as ΔCT = CT(target gene) − CT(a reference gene).

### siRNA knockdown

The siRNA that targeted *GSTM1* was obtained from Origene (SR301988, OriGene Tech, Rockville, MD, USA) with a scrambled non-silencing oligonucleotide as a control. U87 cells were seeded in 6 and 12-well plate with DMEM (10%FBS). The cells were transfected with siRNA (100 pmol) at a confluent density of 70–80% using Lipofectamine 2000 (Thermo Fisher Scientific, Inc.) according to the protocol. The efficiency of knockdown is validated by qRT-PCR.

### Cell growth assay

WST-1, a cell growth assay (TAAR-WBF9, Tools Biotech. Co. Ltd.), was performed to assess the cell viability under knockdown of target gene. In brief, 4,000 cells were plated in 96-well plates and 10 μl of WST-1 was added to each well after knockdown of target gene for 48 h. The relative cell density was obtained with a ELISA reader (absorbance 450 nm, Infinite M200, Tecan Group Ltd., Switzerland) 2 h later after a gentle shake. The optical density (OD) was calculated as: inhibition ratio (%) = (1 − OD value of the experimental group/OD of the control group) × 100.

### Cell cycle analysis

The glioma U87 cells undergoing *GSTM1* knockdown for 48 h were harvested and washed in phosphate-buffered saline, along with scrambled siRNA-treated control cells. The cells were fixed in ethanol and treated with RNase prior to propidium iodide (PI) staining (50 ug/ml). According to the standard analysis protocol, the PI histogram was demonstrated with 5000 cell counts (BD LSRFortessa flow cytometer). FlowJo with Watson Pragmatic analysis was used to analyze the DNA contents of cells.

### Western blot analysis

The cell lysate was denatured and the protein amount were quantified before loading into the polyacrylamide gel. After adequate separation of protein contents by electrophoresis, the gel was transferred to a PVDF membrane. After blocking with 5% skim-milk buffer, overnight incubation with the primary antibody (diluted as suggested in the individual data sheet), followed by a 1:10000X dilution of horseradish peroxidase–conjugated anti–rabbit or anti-mouse antibody (GeneTex, Hsinchu, Taiwan) for one hour, was performed. Signals were detected by using Clarity Western ECL blotting Substrate (Bio-Rad Laboratories, Hercules, CA, US).

### The list of antibodies used

GSTM1(1H4F2) (IHC and western): Novus Biologicals, LLC. (NBP2-22186); STAT3(C-20): Santa Cruz Biotechnology, Inc. (sc-482); Phospho-STAT3 (pSer727) Cell Signaling Technology, Inc. (#9134); p38 MAPK: Cell Signaling Technology, Inc. (#9212); Phospho-p38 MAPK (Thr180/Tyr182): Cell Signaling Technology, Inc. (#9211); NF-κB p65 (C22B4): Cell Signaling Technology, Inc. (#4764); NF-kB phospho-p65 (Ser536)(93HI): Cell Signaling Technology, Inc. (#3033); β-Actin Antibody (C4): Santa Cruz Biotechnology, Inc. (sc-47778).

## Results

### Analysis of ATAC-seq and RNA-seq datasets in GBM patient samples

**Fig. 1:**
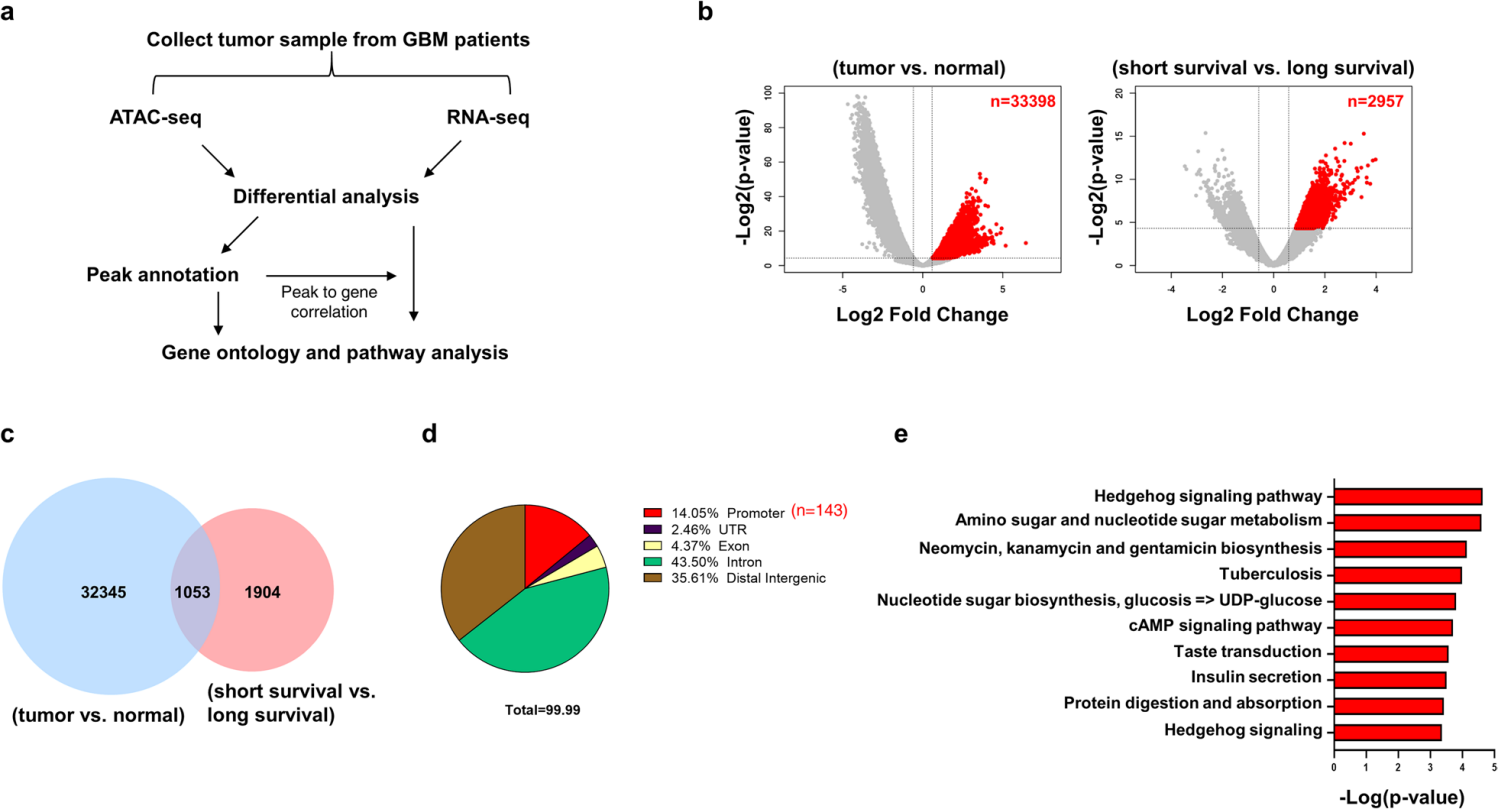
Analysis of ATAC-seq and RNA-seq datasets from GBM patient samples showed the overlapping increased chromatin accessibility within a region and possible pathways involved in GBM tumorigenesis. **a** Flow chart of analysis of ATAC-seq and RNA-seq datasets from GBM patient samples. **b** Analysis of differential chromatin accessibility within the regions showed the significantly increased accessibility within the regions from two different sets of tumor samples (tumor vs. normal and short-survival vs. long-survival). **c** Venn diagrams of overlapping subsets showed the number of overlapping significantly increased accessibility within the regions between these two different sets of tumor samples (tumor vs. normal and short-survival vs. long-survival). **d** Pie plot showed the categorization of genomic regions through annotation of consistently increased accessibility within the regions. *N* = 143 represented the regions that were located in the promoter. **e** KEGG gene ontology analysis of increased accessibility within the regions showed the pathways that may be involved in GBM tumorigenesis

To identify prognostic biomarkers of glioblastoma (GBM) based on chromatin accessibility and differential gene expression, we performed ATAC-seq and RNA-seq on tumor samples collected from GBM patients with short-term (< 0.5 years) and long-term (> 2 years) survival (2 normal tissues and 4 tumor tissues from each of the long- and short-term survival patients were analyzed by RNA-seq and ATAC-seq, while 2 additional normal tissues were analyzed by ATAC-seq) (see the “Methods” section and Table 1 for details) (Fig. 1a). Comparing the chromatin accessibility between these two sets of samples (see “Methods” section), we identified 2,957 regions with significantly increased accessibility (cut off by 1.5-fold change, *p* value < 0.05) in short-survival (chemoresistant; CR) vs. long-survival (chemosensitive; CS) patient samples (Fig. 1b). Among the 2,957 regions, we only focused on 1,053 regions that also exhibited increased accessibility when compared with normal brain tissues (Fig. 1c). To understand the functional implications of these regions, we annotated their genomic features. Among the 1053 genomic regions with increased accessibility, 14.05% were located in the promoter regions of 143 genes (Fig. 1d). KEGG (Kyoto Encyclopedia of Genes and Genomes) enrichment analysis of these 143 genes revealed various pathways, such as the hedgehog signaling pathway (Fig. 1e). In addition to the analysis of ATAC-seq datasets, we also applied differential gene expression analysis by RNA-seq and identified 4,003 and 630 upregulated genes whose expression levels were increased in tumor vs. normal and short-survival vs. long-survival patient samples, respectively (Additional file 1: Fig. S1a). KEGG analysis of increased gene expression in tumor tissues (vs. normal tissues) and short-survival (vs. long-survival) tumor samples showed the pathways of cell cycle and MAPK signaling in these two groups (Additional file 1: Fig. S1b, c). We ranked the fold change of gene expression with significant induction of chromatin accessibility, and the results showed that the expression of most genes was increased with the induction of chromatin accessibility (Additional file 1: Fig. S1d).

### High chromatin accessibility and gene expression in tumor samples from patients with short survival identified *GSTM1* as a putative biomarker

**Fig. 2:**
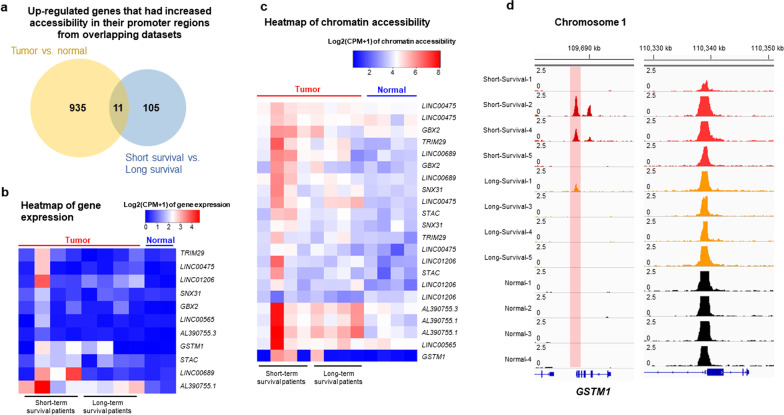
High chromatin accessibility and gene expression by comparing different sets of tumor samples through analysis of ATAC-seq and RNA-seq datasets identified *GSTM1* as a putative biomarker. **a** Venn diagrams of overlapping datasets (tumor vs. normal and short-survival vs. long-survival) showed that 11 genes had induced gene expression and increased chromatin accessibility in their promoters. **b** Heatmap analysis of gene expression showed that *GSTM1* ranked on the top of genes with the highest expression through comparing different groups. Heatmap of RNA-seq data by counts per million (CPM) of each gene was shown. **c** Heatmap analysis of chromatin accessibility of genes showed that *GSTM1* had the highest chromatin accessibility of promoter regions through comparing different groups. Heatmap of ATAC-seq data by counts per million (CPM) of each gene was shown. **d** Comparison of representative ATAC-seq gene tracks in the *GSTM1* locus from short-survival, long-survival, and normal tumor samples. Red-shaded area showed the *GSTM1* tracks from different samples. Gene tracks of *RBM15* locus on chromosome 1 from different samples were shown as controls

To compare the variances of the genomic signature between samples, we performed principal component analysis (PCA) on the ATAC-seq and RNA-seq datasets. The results showed that the tumor samples (short-survival, long survival) were grouped together compared to the normal tissue group (Additional file 1: Fig. S2a). After analyzing ATAC-seq and RNA-seq datasets separately, we combined the analyses of these two datasets by defining the accessible regions on the promoter (Additional file 1: Fig. S2b) and presenting scatter plots between chromatin accessibility on the promoter and gene expression (Additional file 1: Fig. S2c). We found that there were positive correlations between accessible regions and gene expression in both tumor vs. normal brain tissue and short-survival vs. long-survival patients (Additional file 1: Fig. S2c). To consolidate the results from these two datasets, we set the cut off of log2(2) fold change in the ATAC-seq and RNA-seq datasets and the Venn diagrams of overlapping subsets showed that 11 overlapping genes contained higher chromatin accessibility in the promoter regions and higher gene expression both in tumor vs. normal and short-survival vs. long-survival patients (Fig. 2a). A web-based TCGA (The Cancer Genome Atlas) data analysis was applied to these 11 genes, and the glutathione S-transferase mu-1 (*GSTM1*) gene was identified as the most significant gene predicting poor survival in clinical patients (Table 2). Heatmap analysis of gene expression and chromatin accessibility showed that *GSTM1* had the highest chromatin accessibility in tumor samples with short survival times (Fig. 2b, c). The ATAC-sequencing tracks from the genome datasets (short-survival, long-survival, normal tissue) showed high chromatin-accessible regions in the promoter of *GSTM1* in certain short-survival tumor samples (Fig. 2d), consistent with the genomic dataset analysis. The ATAC sequencing tracks from a different genomic locus were shown as a control (Fig. 2d). Positive correlations between chromatin accessibility and gene expression were found in both the promoter and the gene body in *GSTM1* (Additional file 1: Fig. S2d).

**Table 2:** Overlapping of genes containing significantly accessible promoter regions that correlated with upregulated gene expression

Gene_ID	Gene_type	Gene_name	log2FC (ATAC)	*P* value (ATAC)	log2FC (RNA)	*P* value (RNA)	*P* value (TCGA)	Hazard-ratio (TCGA)
ENSG00000134184	protein_coding	*GSTM1*	6.44	0.0001*	8.02	0.0393*	0.022*	1.8
ENSG00000137699	protein_coding	*TRIM29*	2.32	0.0019*	2.33	0.2541	0.28	1.3
ENSG00000144681	protein_coding	*STAC*	1.68	0.0004*	2.91	0.0353*	0.41	1.2
ENSG00000168505	protein_coding	*GBX2*	1.77	0.0094*	4.57	0.0136*	0.11	0.67
ENSG00000174226	protein_coding	*SNX31*	1.57	0.0067*	1.13	0.3342	0.24	0.74
ENSG00000225511	pseudogene	*LINC00475*	2.63	0.0015*	4.6	0.0912	0.038*	1.7
ENSG00000231419	lncRNA	*LINC00689*	2.09	0.0020*	4.48	0.0956	0.16	0.7
ENSG00000242512	lncRNA	*LINC01206*	1.7	0.0125*	5.8	0.0287*	0.56	0.86
ENSG00000260910	lncRNA	*LINC00565*	2.25	0.0168*	1.59	0.3653	0.091	1.5
ENSG00000275830	lncRNA	*AL390755.1*	3.52	0.0001*	4.38	0.0118*	0.89	0.96
ENSG00000287575	lncRNA	*AL390755.3*	2.56	0.0022*	3.55	0.0160*	NA	NA

The asterisk (*) indicated statistical significance (*P* < 0.05) between experimental and control group

### Verification of GSTM1 as a prognostic marker in glioblastoma from a different patient cohort and analysis of TCGA dataset

**Fig. 3:**
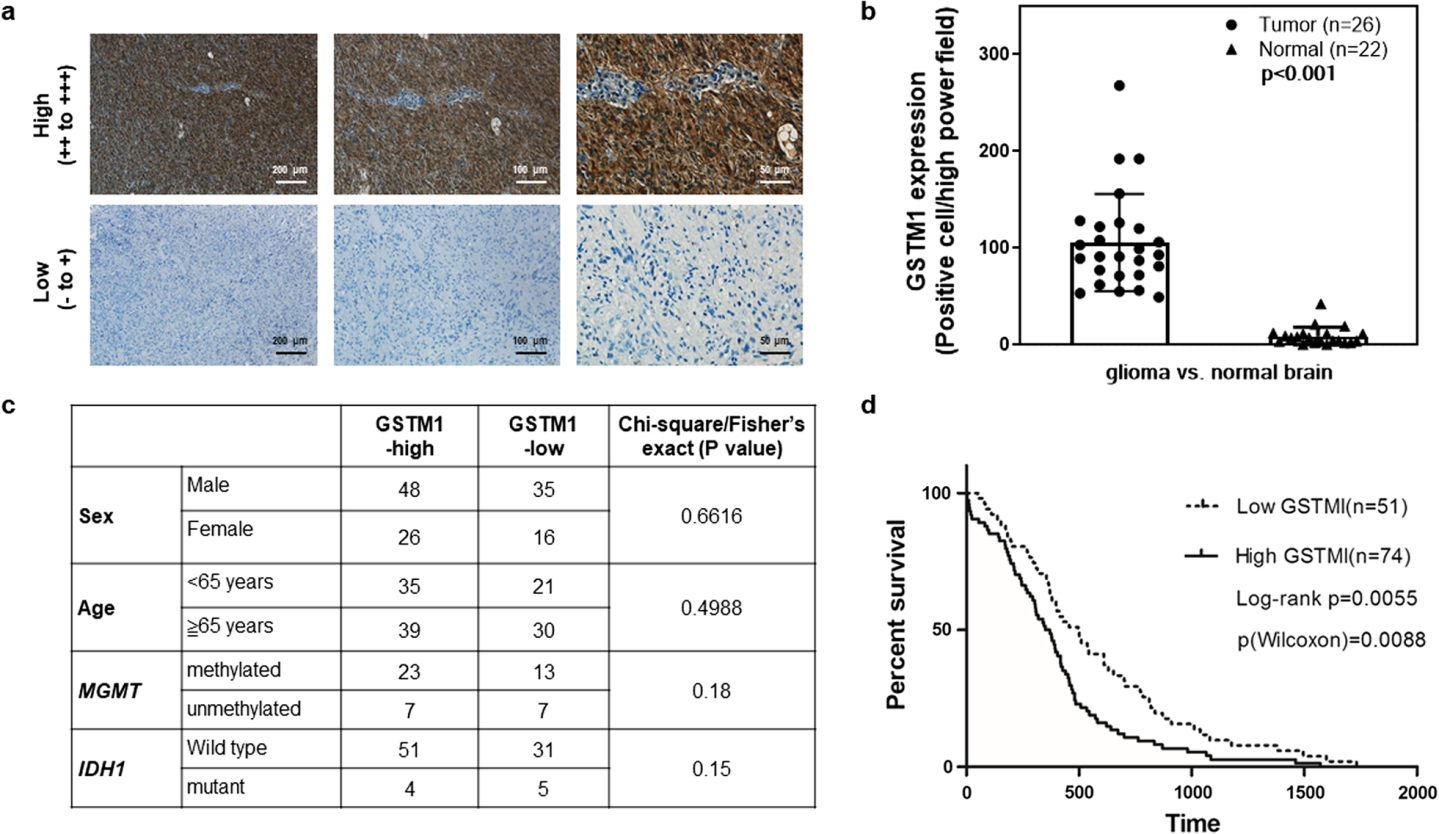
IHC staining, analysis of other genetic markers, and Kaplan–Meier survival analysis identified GSTM1 as a prognostic marker in glioblastoma. **a** A representative IHC staining picture of GSTM1 in GBM tumor samples. The upper panels showed high expression (++ to +++) of GSTM1 under different magnification, whereas the lower panels showed low expression (− to +) of GSMT1. Scale bars were shown in each panel. **b** Bar graph showed the number of GSTM1 positive cells in the tumor tissue vs. normal brain tissue under high power field (A representative picture was shown in Additional file 1: Fig. S2). The numbers of tumor tissue (*N* = 26) and normal tissue (*N* = 22) used were shown. A statistical significance (*p* < 0.001) between tumor and normal tissues was shown. **c** The expression of GSTM1 was not correlated with sex, age, *MGMT* promoter methylation status, or *IDH1* mutation status. **d** A Kaplan–Meier survival analysis of 125 GBM patients according to the expression of GSTM1 showed that high GSTM1 expression was associated with shorter survival (log rank and Wilcoxon analysis)

To further verify the clinical significance of GSTM1 using a different cohort of grade IV glioblastoma patients (*N* = 125), IHC staining of GSTM1 was performed (Fig. 3a). High levels of GSTM1 expression (IHC staining: ++ to +++) compared to low GSTM1 expression (IHC staining: − to +) (see “Methods” section) were defined to compare the GSTM1 IHC staining in GBM samples (Fig. 3a). To measure the extent of GSTM1 expression in the tumor region, direct counting of GSTM1-positive cells in 50 regions from the tumor vs. normal brain region was performed (high power field, 400X) (Additional file 1: Fig. S3). The statistics of high GSTM1 staining were shown in tumor tissues versus normal tissues (Fig. 3b). Seventy-four out of the 125 patients had high levels of GSTM1 expression (++ to +++) compared to 51 patients who had low GSTM1 expression (− to +) (Fig. 3c). To examine the independent prognostic value of GSTM1, further analysis showed that the expression of GSTM1 was independent of other factors, including sex, age, *MGMT* promoter hypermethylation, or *IDH1* mutation (Fig. 3c). Furthermore, the expression of GSTM1 was significantly correlated with overall survival in this GBM cohort (Fig. 3d, *p* = 0.0055 and 0.0088 by log-rank and Wilcoxon test, respectively), indicating that GSTM1 could serve as a biomarker to predict GBM patients’ clinical outcome. Finally, *GSTM1* as a prognostic marker in GBM was corroborated through analysis of TCGA dataset (Additional file 1: Fig. S4a). Interestingly, a search for a downregulated gene biomarker in short-survival patients showed that *ZNF727* expression was repressed and its gene tracks were decreased in short-survival samples (Additional file 1: Fig. S4b–d). TCGA analysis also showed that low expression of *ZNF727* could predict poor overall survival in GBM patients (Additional file 1: Fig. S4e).

### Knockdown of *GSTM1* decreased cell viability and caused cell cycle arrest

**Fig. 4:**
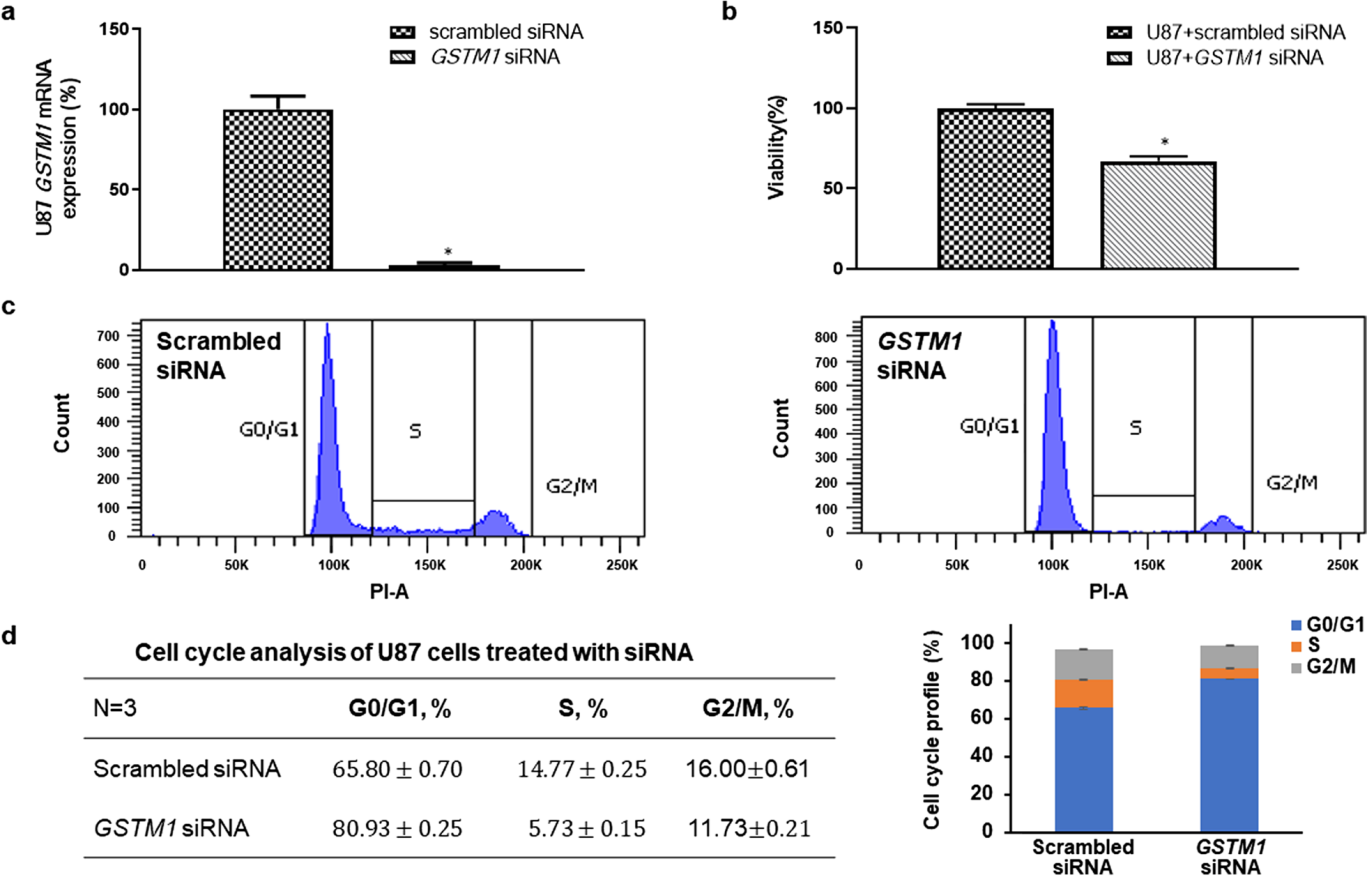
Viability and cell cycle analysis using siRNA-mediated knockdown of GSTM1 showed that knockdown of GSTM1 decreased cell viability and caused cell cycle arrest in U87 cells. **a** Knockdown of *GSTM1* showed a significant reduction of *GSTM1* mRNA expression by RT-PCR assays. Data from three independent experiments were expressed as mean ± s.d. (s.d.: standard deviation). The asterisk (*) indicated statistical significance (*P* < 0.05) between *GSTM1* siRNA and scrambled siRNA groups. **b** Knockdown of *GSTM1* decreased the percentage of viable U87 cells down to 84.3%, compared to control knockdown cells at 48 h. Data from three independent experiments were expressed as mean ± s.d. The asterisk (*) indicated statistical significance (*P* < 0.05) between *GSTM1* siRNA and scrambled siRNA groups. **c** The cell cycle profile showed that knockdown of *GSTM1* caused G0/G1 cell cycle arrest after *GSTM1* was knocked down at 48 h. **d** The percentage of G0/G1 cells was significantly increased in *GSTM1* siRNA knockdown U87 cells compared to scrambled siRNA U87 cells. Data from three independent experiments were expressed as mean ± s.d. A bar graph containing different cell cycle phases (G0/G1, S, G2/M) was shown on the right

To further examine the role of GSTM1 in regulating tumor cell phenotypes, *GSTM1* was knocked down using an siRNA approach in U87 glioma cells. The expression of *GSTM1* was knocked down by 96.8 ± 1.3% as demonstrated by RT–PCR after 48 h (Fig. 4a). Knockdown of *GSTM1* decreased cell viability (down to 67 ± 3% compared to scrambled siRNA control) at 48 h (Fig. 4b). Further analysis of the cell cycle profile showed that knockdown of *GSTM1* caused more tumor cells to be arrested at G0/G1 phase (Fig. 4c, d). The above results indicate that knockdown of *GSTM1* reduces tumor cell viability and causes cell cycle arrest.

### Regulation of the NF-kB and STAT3 signaling pathways by GSTM1

**Fig. 5:**
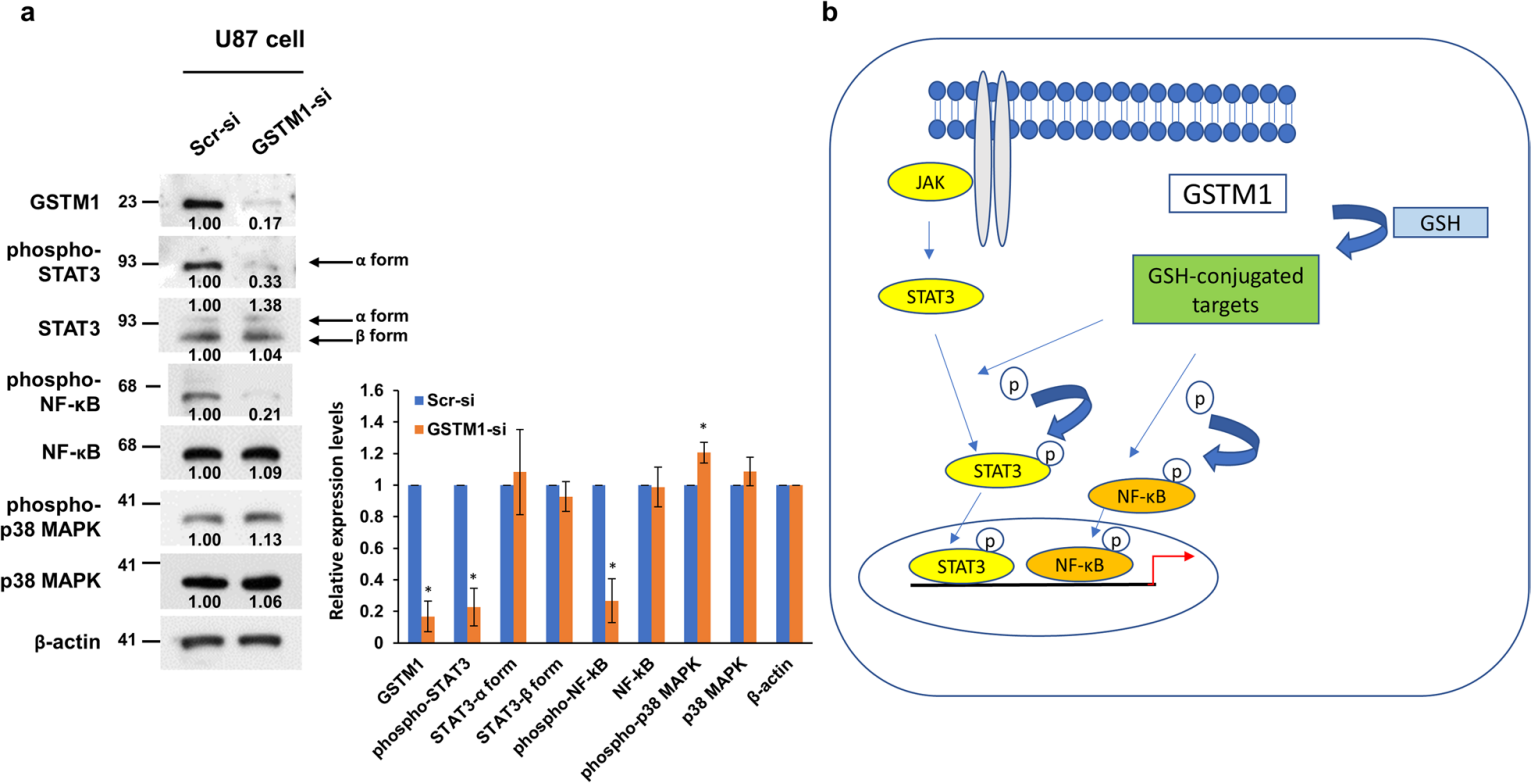
Western blot analysis showed that knockdown of *GSTM1* by siRNA decreased the phosphorylated levels of NF-kB p65 and STAT3 (pSer727) and a model to summarize the results in this report was presented. **a** Western blot analysis showed that knockdown of *GSTM1* in U87 cells at 48 h decreased the phosphorylated NF-kB p65 (phospho-p65) and phosphorylated STAT3 (pSer727) levels, but not the phosphorylated MAP kinase levels. The average levels of proteins from densitomer scanning normalized by β-actin were shown in the bottom of the protein bands. Bar graph showed the levels of different proteins that were quantified and normalized with β-actin by Western blot analysis (triplicate results were shown in Fig. 5a, Additional file 1: Fig. S5). Data from three independent experiments are expressed as mean ± s.d. The asterisk (*) indicated statistical significance (*P* < 0.05) between *GSTM1* siRNA and scrambled siRNA groups. **b** A model summarizes that GSTM1 overexpression induces the STAT3 and NF-kB pathways. The possible mechanism may go through GSH-conjugated targets that subsequently induce the phosphorylation of STAT3 and/or NF-kB p65

Since GSTM1 has been shown to regulate NF-kB signaling [21, 22], we examined whether the NF-kB signaling pathway may be regulated by GSTM1 in U87 cells. Knockdown of *GSTM1* in U87 cells decreased the phosphorylated NF-kB p65 (phospho-p65) levels but not the phosphorylated MAP kinase (phospho-p38) levels (Fig. 5a). Since STAT3 has been shown to be a molecular hub and plays a significant role in GBM tumor progression [24, 25], we further examined whether knockdown of *GSTM1* significantly decreased phosphorylated STAT3 (STAT3-pSer727) levels. The results showed that knockdown of *GSTM1* decreased the phosphorylated STAT3 (STAT3-pSer727) levels, but not the total STAT3 levels (Fig. 5a). This result indicates that GSTM1 regulates both the STAT3 and NF-kB signaling pathways. Whether the regulation of the STAT3 pathway by GSTM1 is direct or indirect remains to be determined.

## Discussion

Classic genetic analyses in glioblastoma have shown that *IDH1* mutation, *MGMT* promoter methylation, 1p/19q codeletion, *G-CIMP* methylation, *BRAFV600E* mutation, and *H3K27* mutation are associated with glioblastoma overall survival [6–9]. However, biomarkers independent of these known biomarkers to predict the survival between short-survival and long-survival GBM patients are unknown [35]. In this report, we selected samples from short-survival (survival < 0.5 years) and long-survival (survival > 2 years) patients followed by ATAC-seq and RNA-seq analyses and identified *GSTM1* as a prognostic marker of GBM, which was confirmed in a different cohort and through TCGA analysis (Additional file 1: Fig. S4a). Since *GSTM1* promoter accessibility and increased expression was only detected in 2 out of 4 short survival patient samples (Fig. 2d), further search for other markers should be mandatory. Following this rationale, similar analysis showed that low expression of *ZNF727* could predict poor overall survival in GBM patients (Additional file 1: Fig. S4b–e). These results suggest that our approach is able to effectively identify prognostic markers that could predict GBM patients’ clinical outcomes.

*GSTM1* methylation has been identified in an integrative analysis of molecular aberrations in GBM genomes [36]. Interestingly, *GSTM1* methylation and *CD40* methylation were coupled under the *G-CIMP* subtype of GBM [7]. Since *G-CIMP* methylation has been linked to a favorable prognosis in GBM patients [7], methylation of *GSTM1* may contribute to a favorable prognosis. Based on the ability of GSTM1 to trigger NF-kB and STAT3 signaling, our results also support the linkage of *GSTM1* overexpression to poor prognosis of GBM patients. In addition to IHC staining of GSTM1 in GBM samples, whether serum levels of GSTM1 could be used as a prognostic marker of GBM patients remains to be determined.

The role of GSTM1 in tumor cell phenotypes has been shown: knockdown of *GSTM1* decreased tumor cell viability and caused cell cycle arrest at G0/G1 phase. The possible pathways regulated by GSTM1 are the NF-kB and STAT3 pathways. In vitro results support the clinical correlation. Our result is also supported by another glioblastoma cohort in which STAT3-pSer727 levels were correlated with clinical outcome [37]. GSTM1 may conjugate reduced glutathione (GSH) to target proteins to further activate NF-kB and STAT3 signaling. Further searches for putative protein targets by GSTM1 should reveal the molecular mechanism of GSTM1-mediated activation of STAT3 and NF-kB pathways. A model of GSTM1-activated kinase pathways is depicted in Fig. 5b.

In conclusion, using ATAC-seq coupled with RNA-seq to analyze datasets from short-survival versus long-survival glioblastoma and tumor versus normal tissues, *GSTM1* was shown to be a biomarker capable of predicting GBM patient survival. Genomic approaches will facilitate the discovery of new biomarkers for glioblastoma patients.

## Supplementary Information

**Additional file 1: Fig. S1.** Analysis of RNA-seq datasets from GBM patient samples revealed the number of upregulated genes and the possibly pathways involved. **a** Differential gene analysis of RNA-seq datasets (tumor vs. normal and short-survival vs. long-survival tumors). The number of upregulated genes (red ink) in different gene sets were shown. **b** and **c** KEGG analysis of increased gene expression in tumor tissues (vs. normal tissues) and short-survival (vs. long-survival) tumor samples showed the possible pathways involved in GBM tumorigenesis in these two patient groups. **d** Ranking of genes with consistently increased chromatin accessibility on promoter through fold change of gene expression showed that more genes with consistently increased chromatin accessibility had increased gene expression through comparing different patient groups. **Fig. S2**. The grouping of tumor tissues (short-survival and long-survival) compared to normal tissues by PCA, the annotation of accessible regions on different genomic locations by pie plot, and the positive correlation between chromatin accessibility and gene expression by scatter plots were shown. **a** PCA showed the grouping of short-survival and long-survival tumor samples together, compared to the grouping of normal samples from ATAC-seq and RNA-seq datasets, respectively. Different colors represented different patient groups. The numbers representing different patients from each category were also shown in Table 1. **b** Pie plot showed that the accessible regions on promoters and other genomic locations from ATAC-seq analysis (see the correlation on Fig. 2b). **c** Scatter plots showed the positive correlation between the chromatin accessibility on promoters and gene expression from different patient groups. The numbers (in red ink) represent the number of genes with increased expression. **d** Positive correlations between chromatin accessibility (promoter and gene body) and gene expression were shown in the *GSTM1* gene from different patient samples. **Fig. S3**. Representative histology of glioblastoma vs. normal brain tissue under IHC staining of GSTM1. **a** IHC of GSTM1 in the glioblastoma tissue: moderate to high expression (++ to +++). **b** IHC of GSTM1 normal brain tissue: low to negative expression (0 to +). **Fig. S4**. TCGA analysis showed the correlation of high *GSTM1* and low *ZNF727* expression with poor overall survival of GBM patients together with gene track analysis of *ZNF727* locus. **a** TCGA analysis showed that high *GSTM1* expression was correlated with poor overall survival of GBM patients. **b** Venn diagrams of overlapping subsets showed that 2 genes had reduced gene expression and decreased chromatin accessibility in their promoters. **c** Heatmap analysis showed the RNA-seq results of the two genes with reduced gene expression and decreased chromatin accessibility in their promoters (*ENOSF1* and *ZNF727*) in different patient samples. **d** Comparison of representative ATAC-seq gene tracks in the *ZNF727* locus from tumors (short-survival, long-survival) vs. normal samples. **e** TCGA analysis showed that low *ZNF727* expression was correlated with poor overall survival of GBM patients. **Fig. S5**. Regulation of the NF-kB and STAT3 signaling pathways by GSTM1. Western blot analysis showed that knockdown of GSTM1 in U87 cells at 48 h decreased the phosphorylated NF-kB (phospho-p65) and phosphorylated STAT3 (pSer727) levels, but not the phosphorylated MAP kinase levels. These data showed the replicate and independently repeated results corresponding to Fig. 5a. **Fig. S6**. Original Western blotting images of Fig. 5a. **Fig. S7**. Original Western blotting images of Fig. S5.

## Data Availability

The datasets used for this report are available from the corresponding author on reasonable request.

## Abbreviations

GBM: Glioblastoma
*IDH1*: Isocitrate dehydrogenase 1
*EGFR*: Epidermal growth factor receptor
*MGMT*: O6-methylguanine-methyltransferase
*G-CIMP*: Glioma CpG island methylator phenotype
ATAC-seq: Assay for transposase accessible chromatin followed by sequencing
GST: Glutathione S-transferase
*GSTM1*: Glutathione S-transferase mu-1
LOH: Loss of heterozygosity
*KDM*: Lysine demethylase
*HDAC*: Histone demethylase
RT-PCR: Real-time PCR
CR: Chemo-resistant
CS: Chemo-sensitive
KEGG: Kyoto encyclopedia of genes and genomes
IHC: Immunohistochemistry
