# Supplementary figures and images for: Chromatin accessibility analysis identifies GSTM1 as a prognostic marker in human glioblastoma patients
Source: Clin Epigenetics. 2021 Nov 3;13:201. doi: 10.1186/s13148-021-01181-8 (PMC8565064; doi:10.1186/s13148-021-01181-8)

**Additional file 1**

**Fig. S1.**

**
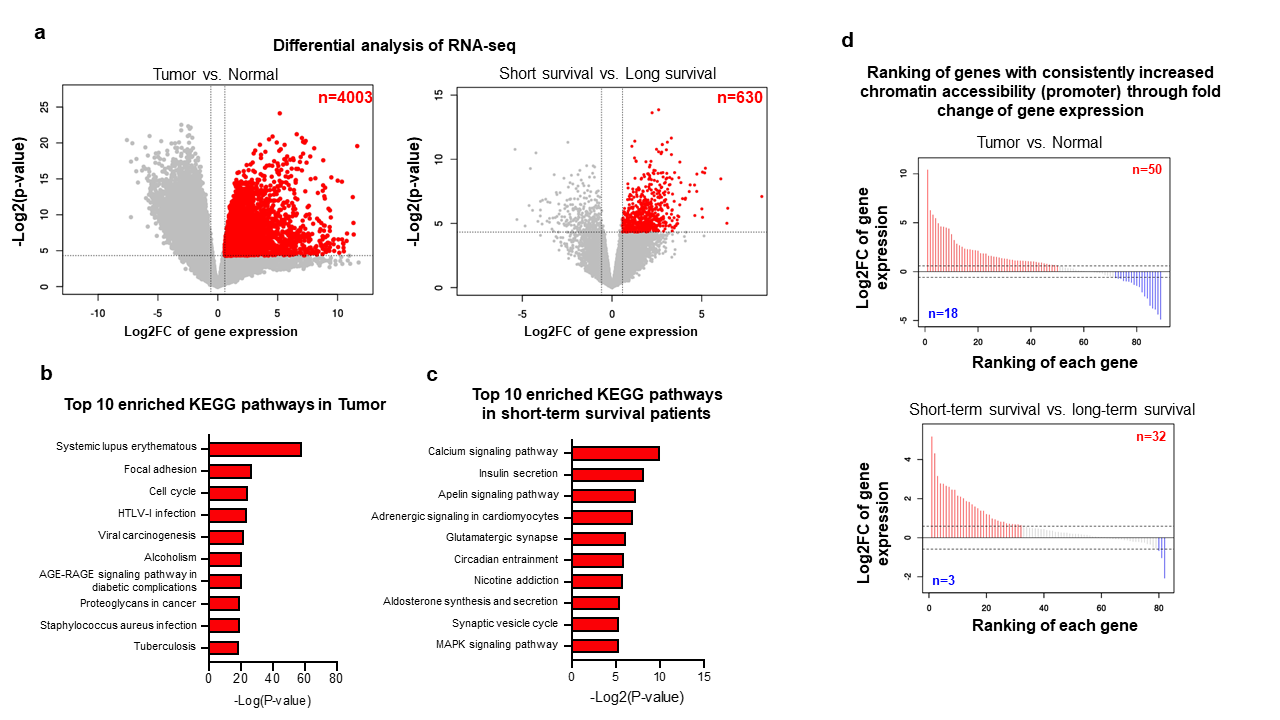
**

**Fig. S2.**

**
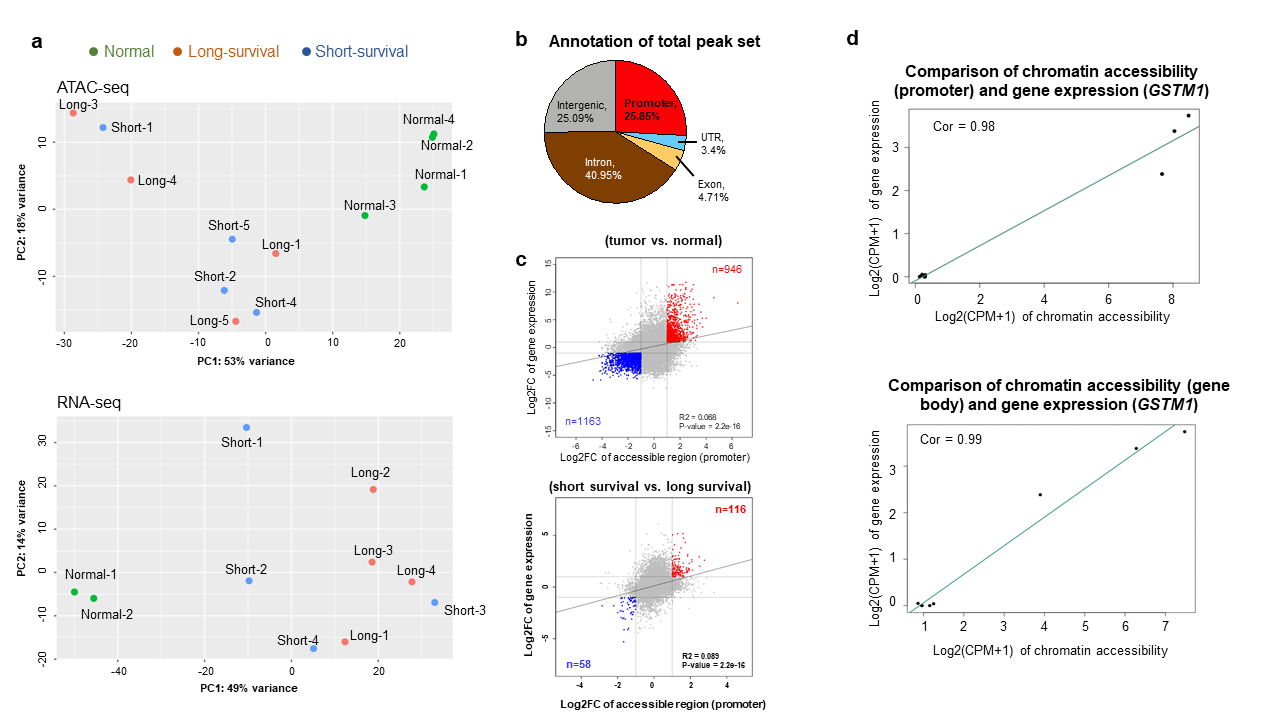
**

**Fig. S3.**


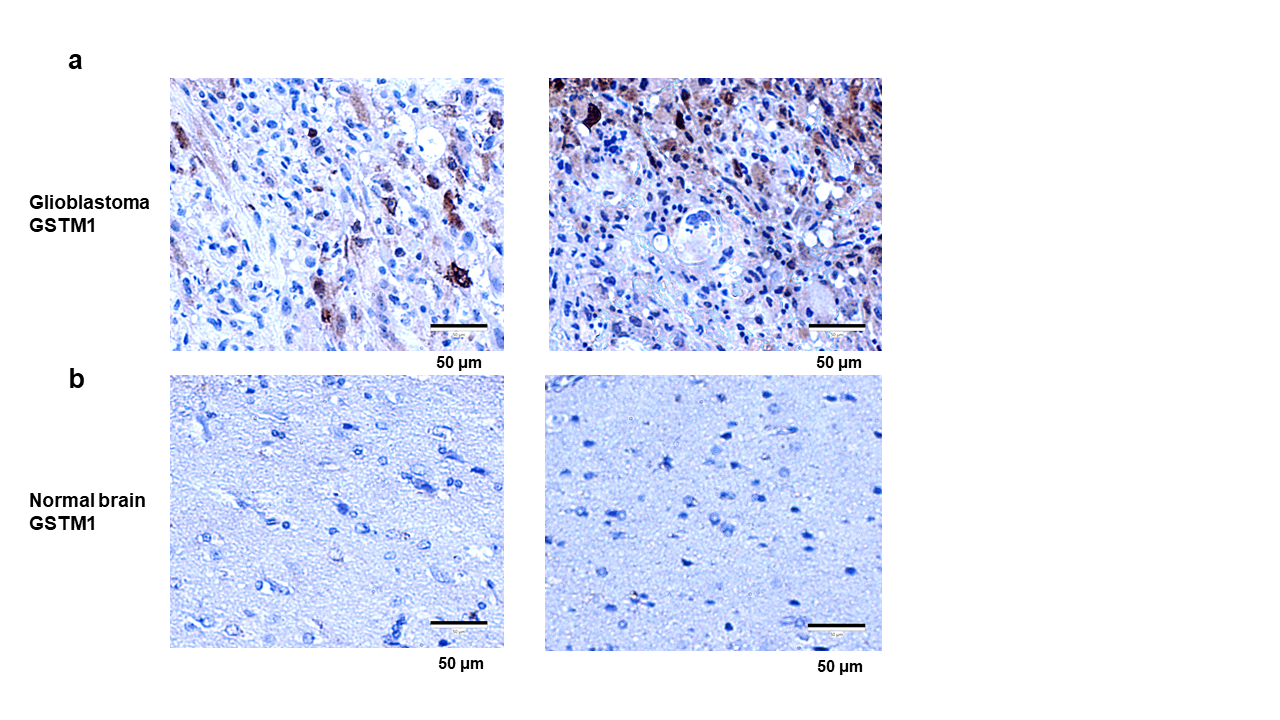


**Fig. S4.**

**
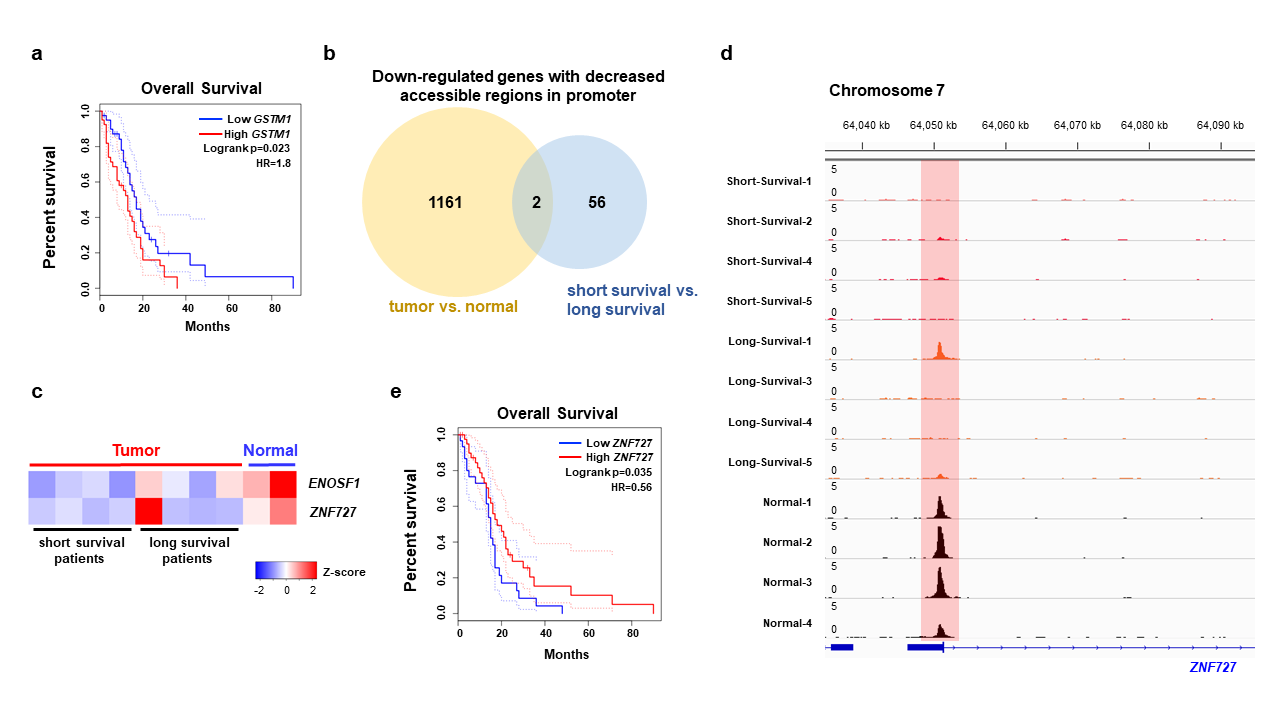
**

**Fig. S5.**


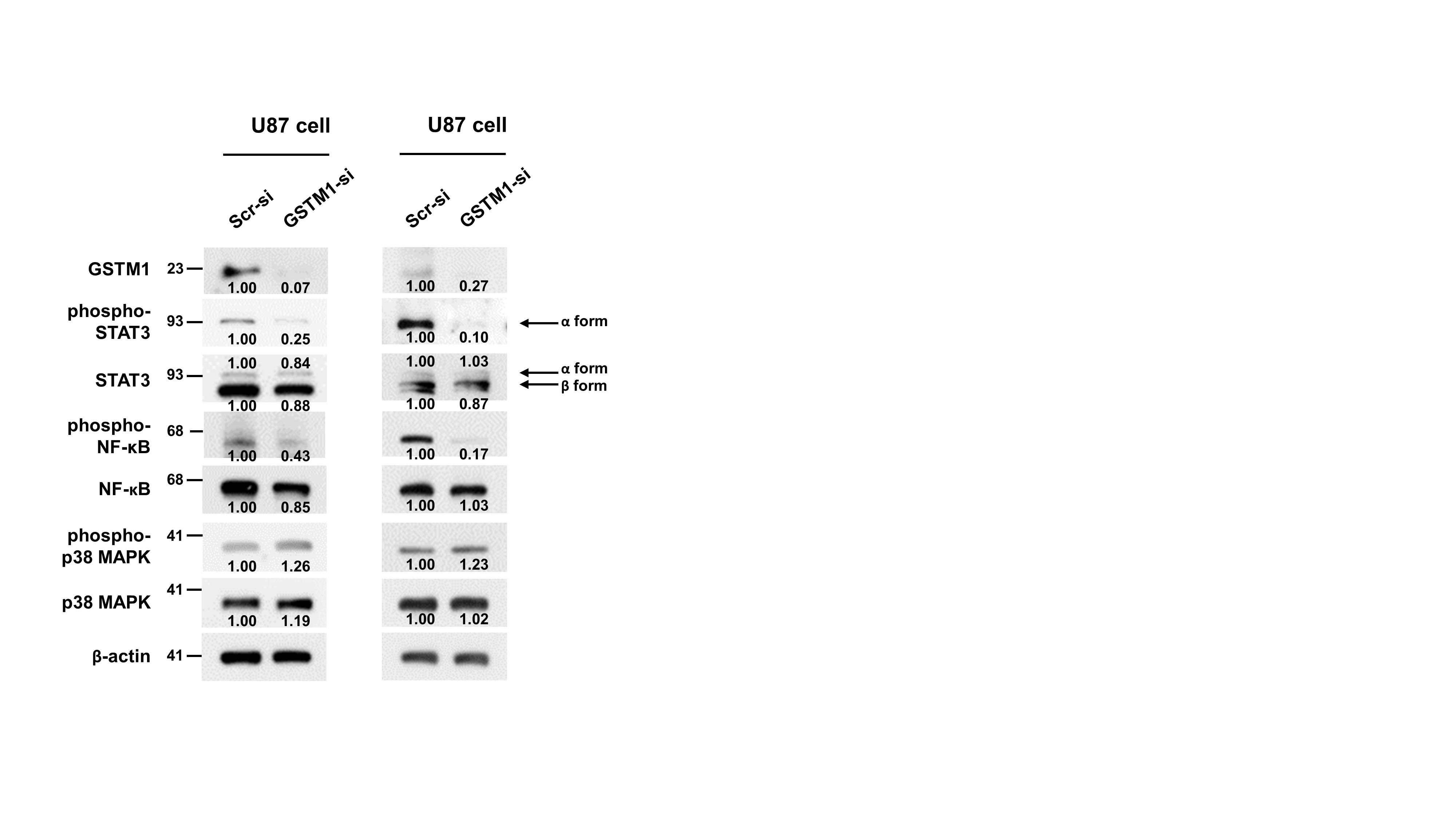


**Fig. S6.**


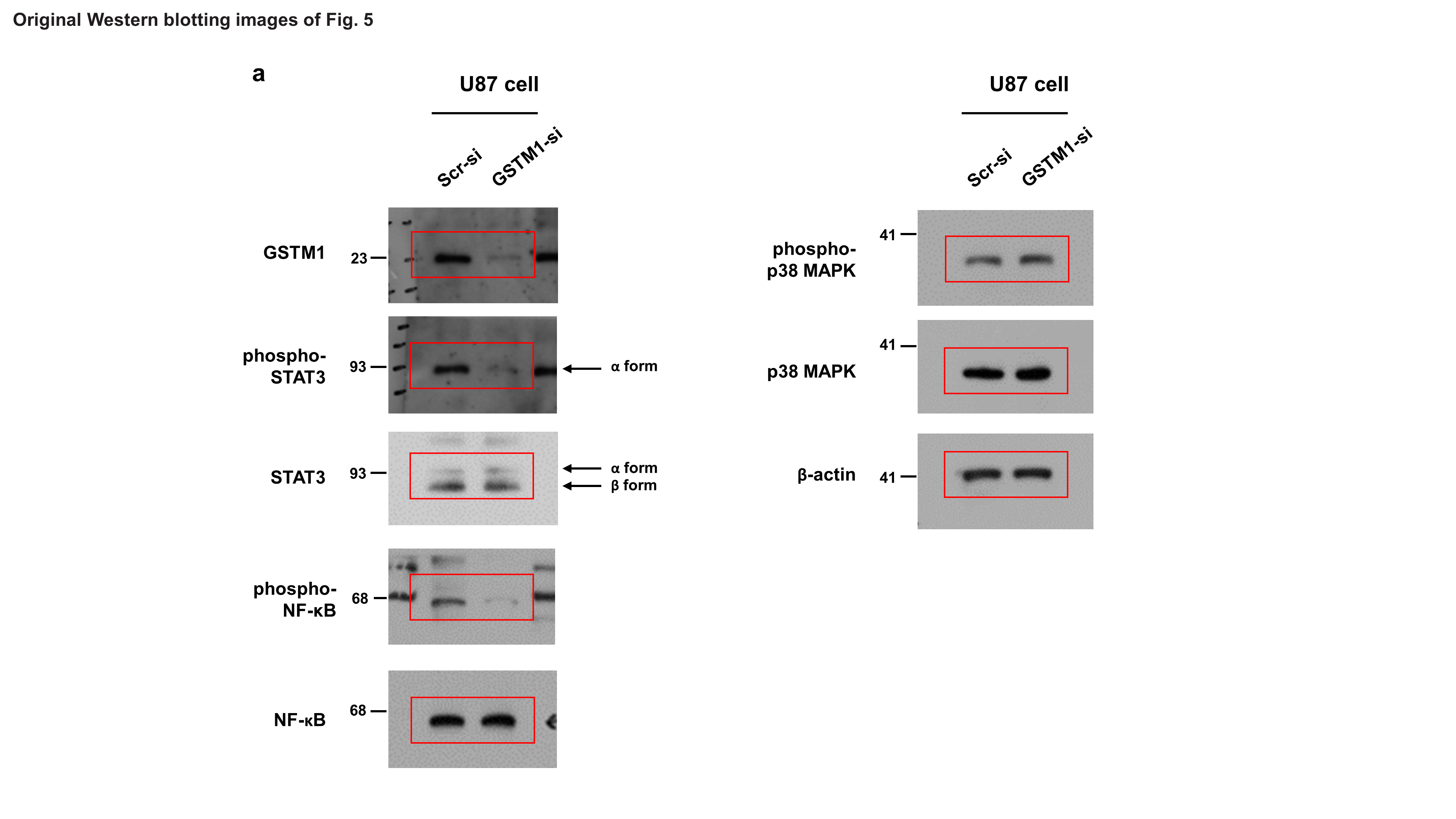


**Fig. S7.**


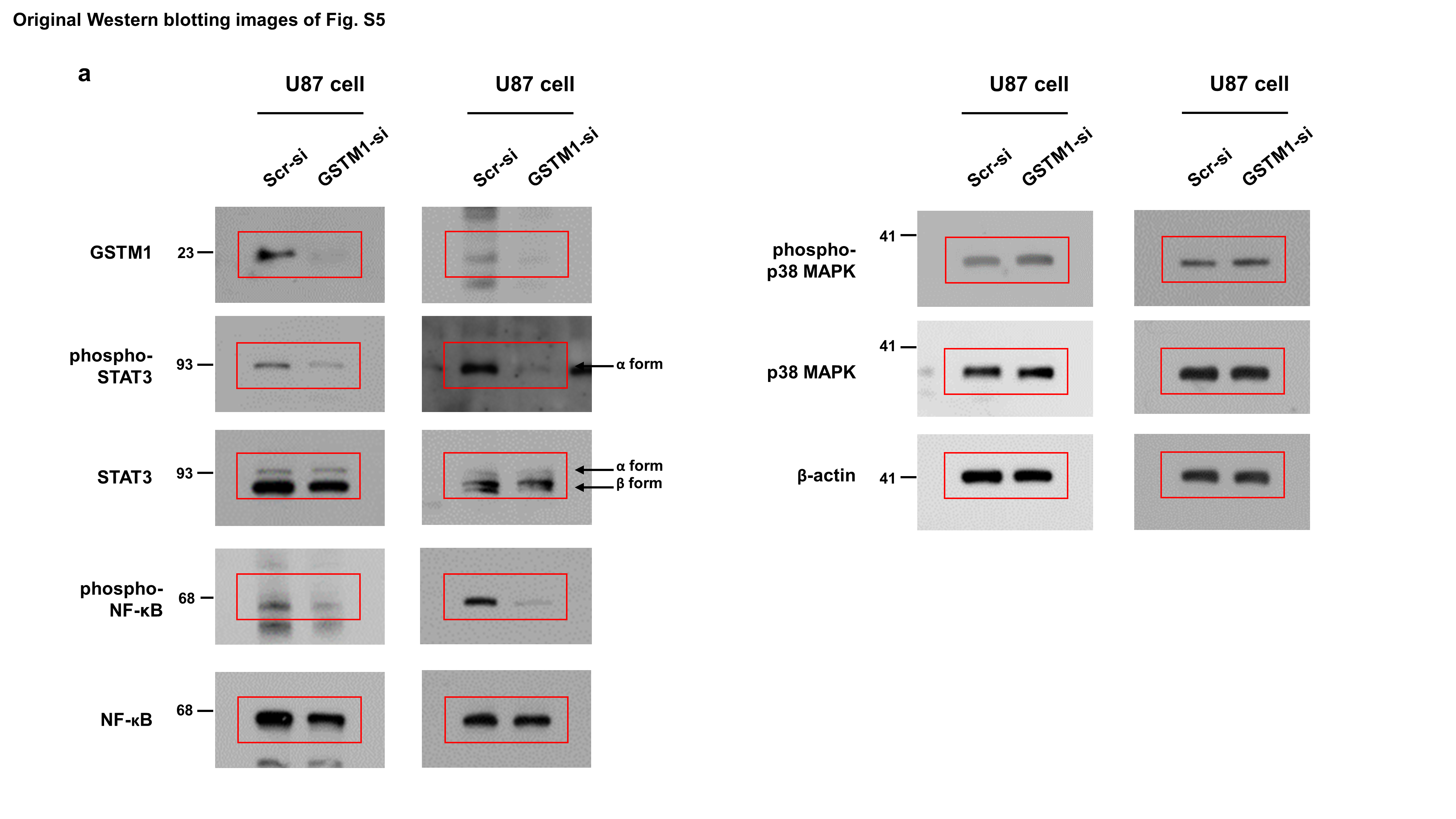

Supplement: Supplementary file 1 — Additional file 1: Fig. S1. Analysis of RNA-seq datasets from GBM patient samples revealed the number of upregulated genes and the possibly pathways involved. a Differential gene analysis of RNA-seq datasets (tumor vs. normal and short-survival vs. long-survival tumors). The number of upregulated genes (red ink) in different gene sets were shown. b and c KEGG analysis of increased gene expression in tumor tissues (vs. normal tissues) and short-survival (vs. long-survival) tumor samples showed the possible pathways involved in GBM tumorigenesis in these two patient groups. d Ranking of genes with consistently increased chromatin accessibility on promoter through fold change of gene expression showed that more genes with consistently increased chromatin accessibility had increased gene expression through comparing different patient groups. Fig. S2. The grouping of tumor tissues (short-survival and long-survival) compared to normal tissues by PCA, the annotation of accessible regions on different genomic locations by pie plot, and the positive correlation between chromatin accessibility and gene expression by scatter plots were shown. a PCA showed the grouping of short-survival and long-survival tumor samples together, compared to the grouping of normal samples from ATAC-seq and RNA-seq datasets, respectively. Different colors represented different patient groups. The numbers representing different patients from each category were also shown in Table 1. b Pie plot showed that the accessible regions on promoters and other genomic locations from ATAC-seq analysis (see the correlation on Fig. 2b). c Scatter plots showed the positive correlation between the chromatin accessibility on promoters and gene expression from different patient groups. The numbers (in red ink) represent the number of genes with increased expression. d Positive correlations between chromatin accessibility (promoter and gene body) and gene expression were shown in the GSTM1 gene from different patien [file 13148_2021_1181_MOESM1_ESM.docx]
